# Editorial: Article Collection on the Human Aspects in Adaptive and Personalized Interactive Environments

**DOI:** 10.3389/frai.2020.606068

**Published:** 2020-11-27

**Authors:** Panagiotis Germanakos, Vania Gatseva Dimitrova, Styliani Kleanthous

**Affiliations:** ^1^ SAP SE, Walldorf, Germany; ^2^ Department of Computer Science, University of Cyprus, Nicosia, Cyprus; ^3^ School of Computing, University of Leeds, Leeds, United Kingdom; ^4^ Cyprus Center for Algorithmic Transparency, Open University of Cyprus, Latsia, Cyprus

**Keywords:** human‐computer interaction, computational intelligence, human factors, personalization, adaptation, user model, algorithm, usability & user experience

The rapid technological advancement and increasing availability of big data in recent years, has transformed computational systems to more dynamic, multidimensional digital communication environments that present highly complex and uncertain information flows, unfamiliar scenarios, situation-specific use cases, and multi-purpose interactions. Such a reality brings more prominently intelligent technologies to the center of attention for providing alternative insights, adaptive interventions, personalized conditions, and smart solutions to the benefit of the unique user. In principle, a vast body of research addresses adaptation and personalization based on ordinary user characteristics (e.g., role, experience, knowledge, interests) or related contextual aspects (e.g., displays, connectivity, processing power). Researchers, professionals, and practitioners in the broader scientific areas of Adaptive Hypermedia, Web Personalization, and User Modeling, have determined numerous user aspects that demonstrate an unquestionable positive influence to the content, functionality, and interactions offered by adaptive and personalized systems in various application fields (e.g., modeling the user preferences and interests for increasing the accuracy of recommender systems, or modeling knowledge and skills in educational hypermedia systems for an enhanced learning experience). Building on this premise, the extraction and use of deeper psychological constructs, values, and abilities (attributes that define individuals, e.g., cognition, intelligence, emotions, personality, expertise), may also fundamentally advance the role of computer-mediated environments that encompass human-computer interactions.

Acknowledging that human-computer interactions are essentially executed on a cognitive level (i.e., users may engage into actions that involve learning, problem solving and decision making), it is of paramount importance to scrutinize and coordinate individual traits and differences throughout the whole design and development process of current practices. Human factors may be exploited during the definition and implementation of the user models or to be regarded as an integrated intelligent component of a system producing smart user interfaces and interaction paradigms. The expected outcomes may offer to users a rich user experience and enhanced usability during the execution of their activities advancing the overall quality of computational systems, services and applications. Nevertheless, considering the multi-dimensional nature of human factors as well as the complexity of the data structures and content meta-characteristics, we could recognize that this is not an easy task. The modeling of individual characteristics and the formulation of respective rules that would guide optimal personalization environments, conditions, and functionality in various contexts and application domains is a long and cumbersome iterative process. In recent years, such individual differences have been extensively explored and utilized in adaptation and personalization systems, yielding in some cases mixed outcomes whereby in others show a significant improvement on the personalization of the user experiences. Thus, there is an indisputable need for a fundamental shift in our understanding of individual differences which considers human aspects inclusively in the design and development process of intelligent solutions. Since, modeling a range of user diversity parameters, e.g. intrinsic human factors, demographics, motivation and self-regulation, and consolidating these in adaptive and personalized systems still remains an open challenge; and viable long-term solutions are yet to be found.

This is especially relevant for systems that promote learning and behavior change which require more holistic human-centered adaptation and personalization. Successful approaches could be realized by a) defining more accurate human-centered models based on intrinsic human factors and abilities, such as perceptual, personality, visual, cognitive, and emotional factors as expressed by the theories of individual differences, as well as on other inherent or more recognizable diversity user characteristics like age, culture, status, motivation, expertise, self-actualization, socio-cultural behavior, etc.; and b) creating intelligent algorithms, interaction principles and smart interfaces that can handle the increasing computational complexity, behavioristic patterns, data structures and the high volume of the generated multi-purpose information.

This article collection is primarily inspired by the International workshop HAAPIE (http://haapie.cs.ucy.ac.cy), held annually in conjunction with the ACM UMAP Conference. It encloses a selection of extended high-quality papers that have been presented in the series of HAAPIE workshops and original unpublished research works that have a considerable contribution and influence in the field. Accordingly, this special issue contains nine contributions discussing interesting ideas in the areas of adaptive information visualization and analytics; human factors and taxonomies; biases and social media; mental/physical health, persuasive technologies and behavior change; e-commerce, motivation and evaluation; learning activity and emotions; and opinion formation on the internet and personality traits. More specifically, Steichen and Fu discuss that cognitive styles have a direct impact when users engage with tasks that include Information Visualizations, and that there are distinctive differences on individual aid choices and preferences, motivating the development of adaptive Information Visualization systems. In the same line of research, Poetzsch et al. argued in their work that data visualizations should be adapted to both the user and the context employing a user model that combines user traits, states, strategies, and actions. They proposed a taxonomy for visualization recommendations paving the way for adaptive data visualizations in analytics. Aïmeur et al. analyzed the motivations and cognitive biases which are frequently exploited by deceptive attackers in Social Network Sites, proposing some countermeasures for each of these biases to provide personalized privacy protection against deceivers. Main concern of Alqahtani et al. was to understand how the persuasive strategies promote mental health. They provide a comprehensive review in the field, and by examining the relationship between mental health apps effectiveness and the persuasive strategies offer design recommendations. In this research direction, Aldenaini et al. provide a systematic review of persuasive technologies for promoting physical activity and reducing sedentary behavior. They answer some fundamental questions in terms of design and effectiveness evaluation, behavioral theories, etc., and reveal the pitfalls and gaps in the present literature. Adaji et al., investigated which factors could tailor persuasive strategies in e-Commerce so to be more effective. They propose the use of shoppers' online shopping motivation in tailoring six commonly used persuasive strategies; showing that persuasive strategies influence e-commerce shoppers differently based on their shopping motivation. Esteller-Cucala et al. discuss five experimentation pitfalls, especially for online controlled experiments (A/B tests), initially identified in an automotive company's website—followed by other sectors, which are highly probable to appear when evaluating personalization features. Alhathli et al. investigates how humans adapt next learning activity selection to learner personality, emotional stability and competence to inspire an adaptive learning activity selection algorithm. The algorithm selects learning activities with varying assumed and taught knowledge adapted to learner characteristics. Lastly, Burbach et al. created an agent-based model and simulated message spreading in social networks to investigate which factors influence whether a user disseminates information or not. Findings reveal that the network type has only a weak influence on the distribution of content, whereas the message type has a clear influence on how many users receive a message.

The accepted manuscripts convey a representative angle of the theoretical dimensions and practical insights when considering individual differences during the process of user modeling, adaptation, and personalization in various research domains and application fields. Their outcomes and suggestions underline the evident potential and capabilities of the related intelligent solution to keep the user *haapie* in the end!

## Author Contributions

All authors listed have made a substantial, direct and intellectual contribution to the work, and approved it for publication.

## Conflict of Interest

The author PG is employed by SAP SE, Walldorf, Germany.

The remaining authors declare that the research was conducted in the absence of any commercial or financial relationships that could be construed as a potential conflict of interest.

## Acknowledgments

This Research Topic is in memory of Prof. George Samaras (1959–2018) who passionately believed that human factors played key role and should be taken into account in adaptation and personalization. Not only was George Samaras a key driver behind initiating the HAAPIE workshop series, he led research that provided some of the early examples of user-adaptive systems that take into account human factors such as cognitive ability and age.

